# 3D Bioprinting of Biosynthetic Nanocellulose-Filled GelMA Inks Highly Reliable for Soft Tissue-Oriented Constructs

**DOI:** 10.3390/ma14174891

**Published:** 2021-08-27

**Authors:** Alexandra I. Cernencu, Adriana Lungu, Diana M. Dragusin, Izabela C. Stancu, Sorina Dinescu, Liliana R. Balahura, Paul Mereuta, Marieta Costache, Horia Iovu

**Affiliations:** 1 Advanced Polymer Materials Group, University Politehnica of Bucharest, 011061 Bucharest, Romania; alex.cernencu@gmail.com (A.I.C.); diana.m.dragusin@gmail.com (D.M.D.); izabela.stancu@upb.ro (I.C.S.); horia.iovu@upb.ro (H.I.); 2 Department of Biochemistry and Molecular Biology, University of Bucharest, 050095 Bucharest, Romania; sorina.dinescu@bio.unibuc.ro (S.D.); roxana.balahura@bio.unibuc.ro (L.R.B.); 3 Horia Hulubei—National Institute for Physics and Nuclear Engineering (IFIN-HH), 30 Reactorului Street, 077125 Magurele, Romania; paul.mereuta@gmail.com; 4 Academy of Romanian Scientists, 54 Splaiul Independentei, 050094 Bucharest, Romania

**Keywords:** 3D bioprinting, methacrylamide-modified gelatin, cellulose nanofibrils, biomaterial inks, 3D nanocomposite scaffolds

## Abstract

Bioink-formulations based on gelatin methacrylate combined with oxidized cellulose nanofibrils are employed in the present study. The parallel investigation of the printing performance, morphological, swelling, and biological properties of the newly developed hydrogels was performed, with inks prepared using methacrylamide-modified gelatins of fish or bovine origin. Scaffolds with versatile and well-defined internal structure and high shape fidelity were successfully printed due to the high viscosity and shear-thinning behavior of formulated inks and then photo-crosslinked. The biocompatibility of 3D-scaffolds was surveyed using human adipose stem cells (hASCs) and high viability and proliferation rates were obtained when in contact with the biomaterial. Furthermore, bioprinting tests were performed with hASCs embedded in the developed formulations. The results demonstrated that the designed inks are a versatile toolkit for 3D bioprinting and further show the benefits of using fish-derived gelatin for biofabrication.

## 1. Introduction

To advance the development of functional materials as biomimetic tissue substitutes, the field of tissue engineering and regenerative medicine recruits the resources, strategies, and technologies from engineering, chemistry and biology, considering both the currently available and pioneering ones. Since the main goal is to achieve highly specialized structures, the research community is inclined to pursue the natural polymers as the most promising outset together with up-to-date fabrication techniques [1]. Recent studies have revealed that 3D printing technology is highly influential in the field of biofabrication due to its flexibility in scaffolds fabrication and its potential of reproducing the 3D complexity of natural tissues [2,3]. In this context, the developments of printing formulations really took off in the last decade and there is still an increased interest in discovering the most appropriate biomaterial ink.

Natural derived biomaterials are favored for the development of 3D printed tissue substitutes, counting on their ability to cope more efficiently with the imposed tissue complexity and they generally include proteins (collagen, gelatin etc.) and polysaccharides (cellulose, alginate, chitosan, hyaluronic acid, etc.) [4,5,6]. Various studies considering tissue-engineered 3D scaffolds have reported the use of the hydrolysates of collagen in bioinks, with gelatin and its derivatives being very encouraging choices for biomaterial fabrication since they express a good interaction with different types of cells (such as fibroblasts, mesenchymal stem cells, adipocytes) and growth factors [7,8,9,10].

The chemical structure of gelatin retains the cell-adherent motifs and closely preserve the biological functions of collagen, regardless of its origin. Gelatin from mammalian sources exhibits thermosensitive behavior and is extensively employed in biomedical applications including 3D (bio)printing [11]. On the other hand, gelatin from fish, which exhibits less temperature sensitiveness and different rheological properties, recently gained interest considering its lower risk of disease transmission and certain religious restrictions. Fish gelatin is considered a reliable alternative to the other types of gelatins, and one can take advantage of ease of dissolution and handling at room temperature to incorporate heat-sensitive constituents [12]. The ability of gelatin to withhold the benefits of collagen provides a considerable leverage over its uses in biomedical applications especially due to its low antigenicity in physiological environment [13]. Gelatin gains tunable mechanical properties through derivatization, when methacrylate side groups are generated on the polymer backbone, aside from the well-acknowledged properties of the parent molecule such as cell-responsive features. Since methacrylamide-modified gelatin (GelMA) can undergo rapid photo-crosslinking in cell-friendly conditions, it became strikingly attractive in bioink formulation and several strategies to preserve the printing fidelity were employed [14]. One method to attain a well-defined deposition of semisynthetic derived gelatin relies on the temperature-dependent sol-gel transition of gelatin, for which mammalian sources are considered [15]. Although the shape fidelity is ensured by cooling the printed structures, this approach may affect sensitive cell types [14]. A different strategy that overcome this issue is given by the use of viscosity enhancers, several studies describing a successful printing when using additives such as gellan gum, alginate, hyaluronic acid, and nanocellulose [16,17,18,19]. The current opinion of the scientific community is still divergent when it comes to which additive is more effective and more conclusive remarks focus on the requirements of target application. Among them, plant-derived nanocellulose has attracted a special interest in the field on 3D (bio) printing due to its rheological properties since in an aqueous solution it exhibits shear-thinning behavior [20]. Numerous research studies have shown cellulose as a biocompatible and low toxic component able to mimic the extracellular matrix (ECM) which further led to an exponentially increasing rate of its use in bioink formulations in the last decade [19]. In recent studies, cellulose was used in the form of oxidized nanocrystals by Xu et al. and nanofibers by Jiang et al. to aid gelatin’s printability and their results highlighted a remarkable enhancement in terms of printability and further a good biocompatibility [21,22]. Furthermore, Ojansivu et al. employed TEMPO-oxidized cellulose nanofibrils (CNF) as a viscosity enhancer for gelatin-alginate based bioink to develop a multicomponent bioink for bone tissue engineering [23]. This study shows that CNF even in small concentration has a highly positive influence not only over the printing process but also over the viability and proliferation of cells. Although the addition of nanocellulose in gelatin-based inks proved to have a beneficial impact on printing and mechanical properties without compromising the viability and proliferation of cells, only two studies have yet considered the use of GelMA instead, for bioink formulation [24,25]. Xu et al. carried out an insightful study on CNF/GelMA ink formulation for extrusion-based printing, studying the molecular interaction between the two components and the influence of the compositional ratio over printability, mechanical properties, morphology, and cytocompatibility [24]. The aforementioned papers employ exclusively gelatin from mammalian sources for the development of biomaterial inks, where CNF plays a crucial role in the printability process. Nonetheless, the stability of printed scaffolds took also advantage of the thermosensitive behavior of mammalian gelatin, whose fast gelation bellow 30 °C additionally provides structural integrity of deposited filament/layer.

Despite the wide use of gelatin from mammalian sources in biofabrication, few studies consider the need for the thermal stability of bioinks at room temperature. In the context of 3D printing, maintaining a precise temperature control over the entire fabrication system is difficult, yet crucial for GelMA. On the other hand, when targeting cells-embedded formulations, the thermosensitive behavior of GelMA is idle.

In this study, we introduced for the first time highly reliable and stable bioinks based on GelMA derived from fish skin in combination with CNF. Fish gelatin grants a superior thermal stability that further surmount the problems associated with significant viscosity changes of mammalian gelatin upon temperature variation. We comparatively studied fish- and bovine-based GelMA biosynthetic formulations by systematically assessing the printability under cell appropriate conditions and following their overall functionality. Acellular hydrogel structures were printed using microvalve-based printing and crosslinked in the presence of adequate photoinitiator. The structural integrity of printed structures was studied aside with the morphological and swelling features of the network. The bioprintability of cell-embedded ink was further assessed in terms of cell viability after printing to demonstrate the benefit of using fish GelMA for biofabrication.

## 2. Materials and Methods

### 2.1. Precursors Chemical Modification

Gelatins were chemically modified according to the protocol described in a previous report [26] with methacrylamide side groups, resulting in a modified gelatin derivative (GelMA) through the direct reaction with methacrylic anhydride in phosphate buffer medium. Methacrylamide-modified gelatins were synthetized from two different sources: gelatin from bovine skin (Sigma-Aldrich gelatin from bovine skin, Type B, Steinheim, Germany), and from fish skin (Sigma-Aldrich-Gelatin from cold water fish skin, BioReagent, Steinheim, Germany) to a methacrylation degree (%MD) of 25% and 33%, respectively. The %MD of GelMA was evaluated using ^1^H-NMR as previously described by Hoch et al. and Wang et al. [27,28]. About 20 mg of pristine and modified gelatin was dissolved in 0.75 mL of deuterium oxide (99.9 atom %, from Sigma Aldrich (Steinheim, Germany) and the ^1^H-NMR Spectra were registered on 600 MHz Spectrometer (Bruker, Ascend^TM^ 600, Rheinstetten, Germany). To calculate the %MD, the amine signals of methacrylamide-modified lysine were normalized by setting the phenylalanine signals as the internal reference and the following relation was used:

(1) $$\%\mathrm{MD}\;=\left(1-\frac{\mathrm{Lysine}\;\mathrm{integration}\;\mathrm{signal}\;\mathrm{of}\;\mathrm{GelMA}}{\mathrm{Lysine}\;\mathrm{integration}\;\mathrm{signal}\;\mathrm{of}\;\mathrm{Gelatin}}\right)\times 100$$

Never-dried bleached-kraft pulp from softwood (kindly supplied by StoraEnso^TM^) was used for the preparation of the aqueous CNFs. According to a previous report [29], aqueous CNFs (1.10% *w*/*v*) was synthetized at pH 10.5 using the TEMPO/NaBr/NaClO oxidation system, with 2.5 mmol g^−1^ cellulose of NaClO. The total acidic content of CNFs was quantified using conductometric titration as 835 µmol/g. The reagents employed in the modification treatment of cellulose such as TEMPO (free radical, 98%), Sodium bromide ACS (≥99%), Sodium hypochlorite solution (NaClO) (12% active chlorine) EMPLURA^®^ (Steinheim, Germany), Sodium hydroxide 98% (pellets) and hydrochloric acid ACS (37%) were reagent-grade chemicals purchased from Sigma-Aldrich (Steinheim, Germany). Lithium phenyl-2,4,6-trimethylbenzoylphosphinate (LAP) (≥95%; crystalline powder) and Dulbecco’s Modified Eagle’s Medium (DMEM) were also purchased from Sigma-Aldrich (Steinheim, Germany).

### 2.2. Bioink Formulation and Scaffold Design

A series of ink formulations were designed as aqueous blends of CNF and GelMA (derived from either bovine skin: B-GelMA or fish skin: F-GelMA) in weight ratios of 1:10 and 1:20 respectively and will be further referred to as B11 and B12; F11 and F12 as described in Table 1, where 1.1 CNF *w/v* % was used to prepare all samples. The dissolution of the corresponding amount of gelatin (based on the dried weight of CNFs) into the CNF gel was carried out using an ultrasonic water bath at 37 °C. An aqueous solution of LAP (25 mg/mL) was prepared, sterilized using 0.2 µm filter, and added to the formulation to a final concentration of 0.3% (wt/vol). For the bioprinting tests, each ink formulations containing initiator was mixed with human adipose-derived stem cells (hASCs) in a concentration of 1 × 10^6^ cell/mL.

### 2.3. Acellular Scaffold Fabrication and Printability Assessment

The printing tests and the scaffolds fabrication were performed using the microvalve-based bioprinting process (3D Discovery bioprinter RegenHU, Villaz-St-Pierre, Switzerland). First, for as much as F-GelMA and B-GelMA exhibit different rheological behavior regarding temperature changes, line-printing tests were performed at temperatures in the range of 25–40 °C. In the interest of a fair comparison between the two types of GelMA, the printing process of the multiple-layer constructs was carried out at 37 °C for all formulations. The biomaterial ink was inserted in a 3 mL cartridge and mechanically stirred using the cartridge cell agitator for 15 min for the material to reach steady state. The 3D testing samples were designed as a disc (10% honeycomb pattern infill), square (10% grid-shape infill), and hollow triangle with dimensions of 10 mm and thickness of 4, 10, and 20 layers, respectively. BioCAM^TM^ software (Version 1.0, RegenHU, Villaz-St-Pierre, Switzerland) was used to generate the G-code file/printing protocol and the 3D structures were printed layer-by-layer on glass plates with the temperature controlled microvalve-based print-head using a contact dispensing microvalve (CF300 ID = 0.3/S = 0.1) and needle with an inner diameter of 0.3 mm (CF300 ID = 0.3/L = 2.4). The biomaterial inks were printed layer-by-layer and crosslinked by exposing each layer to UV (365 nm) for 30 s. The fabrication parameters (pneumatic pressure, valve opening time, printing speed) were optimized for each formulation to ensure a continuous filament deposition and further the printability of the biomaterial inks as well as the stability of printed 3D-structures were studied in a qualitative manner.

To preserve and stabilize the structure and shape of the pores for further characterization, the printed acellular hydrogel scaffolds were refrigerated in ultra-low temperature refrigerator at −80 °C and then freeze-dried for 6 h.

### 2.4. Characterization of Physical Properties

#### 2.4.1. Morphology

The morphology of the freeze-dried acellular printed scaffolds was assessed by using a Scanning electron microscope (SEM) produced by Zeiss, model EVO MA15, Cambridge, United Kingdom. The images were obtained in variable pressure mode using secondary electrons (VPSE) and beforehand the samples were sputter-coated with a fine layer of gold using a bench top evaporator in vacuum (Quorum Technologies, Cambridge, United Kingdom). For all the samples the pressure was around 40 Pa, the accelerating voltage was 12 kV, with a probe current of 90 pA and the magnification was 250× at a working distance in the range 8–14 mm.

#### 2.4.2. Swelling Behavior

The hydrophilic character of the 3D freeze-dried samples was determined by monitoring the swelling process in DMEM mimicking physiological conditions (pH 7.4 and 37 °C). Three specimens out of each composition were first weighted (W_d_), then placed in DMEM under mild shaking and the wet weight of the samples (W_i_) was recorded over time up to 24 h. The swelling degree (SD %) for each sample was calculated according to the following equation:

(2) $$\mathrm{SD}\;\left(\%\right)=\frac{W_{i}-W_{d}}{W_{d}}\;\times \;100$$

While the maximum swelling degree (MSD %) for each sample was similarly calculated considering the wet weight of the samples at equilibrium, the average values were reported with error bars depicting standard deviation.

### 2.5. Evaluation of Cellular Response

Human adipose-derived stem cells (hASCs) were cultured in DMEM, supplemented with 10% fetal bovine serum (FBS) and 1% antibiotics, and expanded up to passage 6, then used for biocompatibility studies: (i) in direct contact with the 3D printed scaffolds; (ii) embedded in the material ink forming a bioink. (i) Acellular scaffolds biocompatibility. hASCs were seeded on the printed scaffolds, allowed to adhere for 24 h. The resulting 3D cultures were moved to new wells and maintained in culture in complete media for up to 7 days in standard culture conditions (37 °C, 5% CO_2_ and humidity). (ii) Evaluation of cell’s viability toward 3D biofabrication. 1 × 10^6^ hASCs/mL were embedded in material ink, and the resulted bioink was printed using the same parameters as for the acellular scaffolds. The 3D bioprinted scaffolds were washed in sterile PBS to remove any unreacted components and then maintained in complete culture media for 7 days in standard conditions. For both acellular and cellular embedded scaffolds 3D, cell viability was measured by quantitative MTT assay, and cytotoxicity was evaluated using LDH assay (In Vitro Toxicology Assay Kit, Lactic Dehydrogenase based, Sigma-Aldrich). The biological evaluation was performed after 24/48 h and 7 days of cells culture. The ratio between live and dead cells was assessed by fluorescent label with LiveDead assay (LIVE/DEAD™ Viability/Cytotoxicity Kit, for mammalian cells, Thermo Fisher Sci, Waltham, MA, USA and visualized through confocal microscopy (Nikon Eclipse A1, Nikon, NY, USA).

### 2.6. Statistical Analysis

All experiments were performed in triplicate. Statistical analysis was performed in GraphPad Prism 6.0 by using one-way ANOVA method and Bonferroni correction. Statistical significance was considered for *p* < 0.05.

## 3. Results and Discussion

### 3.1. Precursors Chemical Modification

**Figure 1:**
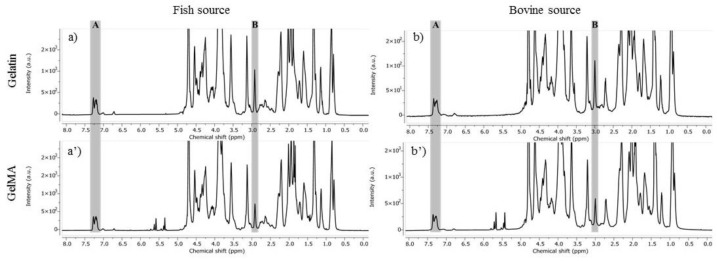
^1^H-NMR spectra of gelatin (**a**,**b**) and GelMA (**a**’,**b**’) from different sources: (**A**) denote the phenylalanine signal of gelatins used as the internal reference and (**B**) denotes the signal of the amino groups in lysine residues used to calculate the %MD.

The prepared F-GelMA and B-GelMA are chemically modified gelatins where methacrylamide functional groups are formed at the free amino groups in gelatin upon reaction with methacrylic anhydride. Thereon, GelMA may undergo crosslinking via photopolymerization in the presence of water-soluble initiators. The methacrylation degree (%MD) describes the ratio of photo-reactive sites with respect to the total amino groups in precursor gelatin determining thus the crosslinking extent of GelMA-hydrogel network. As a consequence, the mechanical properties, swelling behavior and cells viability are strongly influenced by the %MD value [8]. In this research, the %MD of F-GelMA and B-GelMA were assessed using ^1^H-NMR Spectrometry (Figure 1. The new methacrylamide protons identified at 5.72–5.67 and 5.46–5.43 ppm in the spectra of GelMA confirm the success of the modification reactions. Considering the phenylalanine signal as the internal reference at 7.2–7.4 ppm (A—in Figure 1) the two-integration signal of amino groups in lysine residues at 2.8 ppm were compared (B—in Figure 1). The ^1^H-NMR data were quantified and the %MD for F-GelMA and B-GelMA were determined at 25% and 33% respectively.

### 3.2. Biomaterial Ink Characterization and Printability Assessment

The bioprinting process rises serious challenges in terms of materials choice since it fundamentally imposes high water content within the bioink formulation. CNFs comes in aid as a structural builder since it has already been shown to improve the rheological behavior of inks through hydrogen bonding and hydrophobic interactions [30]. The dissolution of GelMA within CNF suspension resulted in homogeneous biomaterial inks. Based on gelatin source and GelMA/CNFs ratio, the formulations were comparatively studied by systematically assessing their printability. According to previous studies, GelMA/CNF formulations exhibited shear-thinning behavior and sol-gel transition, which is required for the extrusion-based printing method [24,31]. Also, the importance of controlling shear-stress in micro-valve-based system has been investigated by other researchers, and their study found that it is crucial in balancing printing resolution and cell integrity [32]. Considering that both the type and amount of GelMA within biomaterial ink formulation induce different rheological behavior, significant adjustments of printing parameters are required in order to optimize the printing process for peak performance.

**Figure 2:**
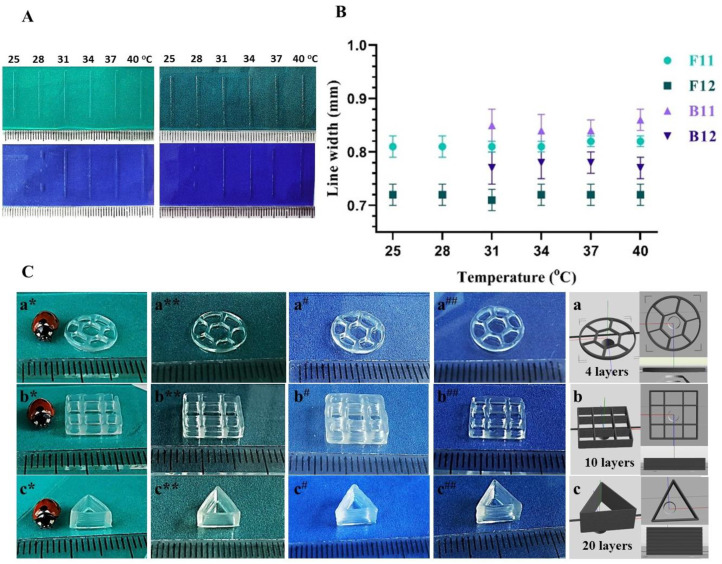
(**A**) Photographs of line-printing tests performed by varying the temperature from 25 to 40 °C. (**B**) Measurements of printed line widths vs. temperature. (**C**) Representative 3D printed constructs with F11 (*), F12 (**), B11 (#), B12 (##) designed as disc (10% honeycomb pattern infill) (**a**), square (10% grid-shape infill) (**b**) and hollow triangle (**c**) with dimensions of 10 mm in 4, 10, and 20 layers, respectively.

The printing pressure, valve opening time (VOT), and nozzle diameter are the three main fabrication parameters that govern the filament deposition in a microvalve-based bioprinting system. Nonetheless, for thermo-responsive inks, temperature becomes one of the main factors that affect the printing process. Although they bring the benefit of rapid gelation during printing, thermo-responsive inks are narrowing the printability window to a specific temperature range. Line-printing tests performed by varying the temperature from 25 to 40 °C depicted along with the measurements of printed line widths in Figure 2 (A and B respectively), demonstrate the printing behavior of formulated inks with respect to temperature changes. As it can be observed in Figure 2A, F11 and F12 allow a uniform and continuous filament deposition regardless of increasing temperature. The results showed that the viscosities of F11 and F12 biomaterial inks are not influenced by temperature to a sufficient extent to affect their printability. Since F-GelMA achieves thermal stability above 10 °C [28], combined with CNF, F11, and F12 showed a very reliable and stable filament deposition. In contrast, it can be observed that the printing with B11 and B12 formulations is highly influenced by temperature, since B-GelMA requires temperatures above 30 °C to become liquid and thus printable [28]. Regardless of operating pressures and feed rate, at room temperature, neither B11 nor B12 generate a continuous filament deposition since the formulations are fully or partially gelled. Above 30 °C, B-GelMA based inks become fluid and they exhibit constant filament widths. It is apparent that, in comparison with F11 and F12, the printability window of B11 and B12 is significantly narrower since printing temperatures between 30 and 40 °C are imposed. An overview of the printing parameters used for each ink formulation is given in Table 2.

When using contact printing (microvalve-based printhead), the printing process is mainly controlled by VOT, and the minimum pressure is required. Nonetheless, the deposition of more viscous inks still demands higher pneumatic pressures. Considering that the applied pressure strongly influences the cellular behavior, the printing process was optimized herein by trial and error to minimum pressures for each composition by combining the pressure with longer VOT. Therefore, by tuning the VOT between 600 and 1000 µs, the applied pressure required is in the range of 40 to 70 kPa, thus obtaining filament widths roughly around 0.8 mm. Following the tabulated data, it can be noticed that for F-GelMA based inks a longer VOT is needed to generate the uniform material deposition with increasing the amount of GelMA: 650 µs for F11 compared to 800 µs for F12. With respect to applied pressure, it can be noticed that beside a longer VOT, no additional pressure is required for printing with F12 formulation. In microvalve-based printing, as soon as an ink exceeds the printability range, a higher pressure will not contribute to filament formation [33].

By increasing the GelMA concentration from 11% to 22%, only slight changes in printing parameters are required which suggests that F-GelMA based formulation more readily exceed the range of printability at low pressures and further exhibit a large printability window. In contrast, for dispensing process of B-GelMA based formulations, longer VOT and higher pressures are required: 900 µs and 50 kPa for B11 and 1100 µs and 70 kPa for B12 respectively. In this case, high amount of GelMA imposes a considerable increase of VOT and printing pressure that suggests significant changes in viscosity with increasing GelMA concentration. Ink formulations with increased viscosity require higher pressures that may further affect cell viability [34], narrowing its uses in biofabrication. The longer VOT and higher pressures required for B-GelMA inks deposition suggest that B11 and B12 are both more viscous that F12. Hence, considering its low viscosity, F-GelMA formulations seem to be more appropriate for cell embedding.

In Figure 2B it can be observed that under optimal printing parameters the filament widths tolerably vary with temperature. Although CNF significantly improve the printability of GelMA, whose dispensing is unachievable at low concentrations and constant temperature, the CNF aqueous suspension exhibits material post-extrusion swelling. The addition of GelMA seems to reduce the post extrusion swelling and even thinner filament are obtained with increasing protein concentration. Thus, slower filament elongation is observed for inks with higher concentration of GelMA, where filaments of 0.72 ± 0.02 mm and 0.78 ± 0.05 mm for F12 and B12 respectively, are obtained. Although the printed line width measurements denote differences with respect to both type and amount of GelMA, these differences tend to become minor at a larger scale.

Considering the thermal sensitive behavior of B-GelMA, all 3D scaffolds were printed at 37 °C for a fair comparison of B-GelMA with F-GelMA formulations. Figure 2C depicts 3D constructs printed with developed formulations with an increasing number of layers from 4 to 20. It is illustrated herein that all 3D constructs exhibit high shape fidelity and well-defined internal structure. Yet, by comparison, a slightly higher printing resolution can be observed when printing with F12 and B12. In the course of this experiment, optimization of the process for high resolution microvalve-based printing played an important role in providing 3D accurate models. The addition of CNF to GelMA is sufficient to prevent the 3D printed filaments from collapsing during printing. We have validated that using CNF in combination with F-GelMA produce comparable results with previous studies that employ mammalian sourced gelatins [24,25] Further, the stability of the strands is strengthened by concomitant UV-crosslinking so that the printed constructs display accurately nice lines regardless of the number of layers. This approach leads to results that go beyond previous reports which employ post-printing crosslinking of CNF/GelMA inks [24,25]. The print quality of our hydrogel-based materials showed not to decrease as the layer count rises and no deformation of the first layer can be observed even in scaffolds build out of 20 subsequent layers (Figure 2C). The fabrication of scaffolds as different model designs demonstrates the ability of the formulations to withstand their shape in different patterns and heights and illustrates how the viscoelastic properties of CNF improve printing. In combination with CNF, F-GelMA inks exhibit at same concentration similar printing resolution as B-GelMA inks under cell appropriate conditions. Nonetheless, the themosensitive character of an ink significantly restricts its uses in biofabrication processes, since just a precise temperature control will ensure the reproducibility. On that account, thermally stable F-GelMA inks yielded significant speed advantages when it comes to ink preparation (ease of protein dissolution), process optimization, more reliable and stable material deposition, thus providing a wider applicability range.

### 3.3. Acellular Scaffolds Characterization

**Figure 3:**
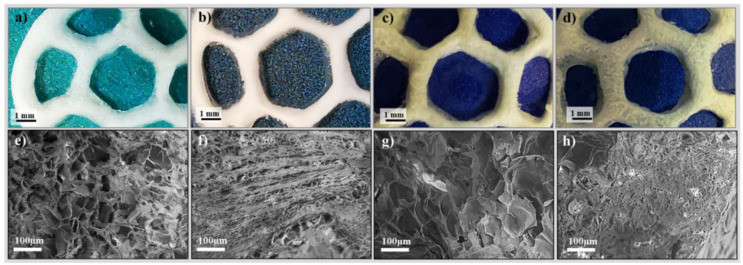
Representative digital photographs (**a**–**d**) and SEM images (**e**–**h**) of freeze dried 3D scaffold printed with F11 (**a,e**), F12 (**b,f**) B11 (**c,g**) and B12 (**d,h**).

Considering the major role of the macro- and micro- internal architecture in overall performance of the scaffolds, including the behavior and interactions of cells within the material, the morphology of freeze-dried acellular scaffolds was investigated. The photographs and SEM images of lyophilized 3D printed discs are shown in Figure 3. On the account of high-water content within the formulation (approx. 80%), highly porous structures were obtained through freeze-drying. The originally printed model is maintained to a reasonably extent after drying, but it can be observed that the shape of pre-designed infill pores does not straightly align in the hexagonal pattern (Figure 3a–d). The SEM micrographs of printed acellular scaffolds (Figure 3e–h) capture the characteristic morphology of hydrogels featuring different structuration of the solid network along with the distinctive size distribution of vacated pores with respect to solid content. Porous structures with pores of irregular shapes were observed in both F-GelMA and B-GelMA based materials. Scaffolds with loose structures and pore sizes up to 100 µm were formed in samples with a CNF/GelMA ratio of 1:10 (Figure 3e,g). With increasing the protein content, the pores appear to decrease in size as a much tight microporous structure can be observed for samples with a 1:20 CNF/GelMA ratio. F12 scaffold (Figure 3f) exhibits a very compact structuration where pores of sizes below 30 µm are homogenously distributed. On the other hand, the internal architecture depictured from B12 scaffolds (Figure 3h) illustrates in addition to densely packed small pores also below 30 µm, some gaps in structuration where pores of 80 ± 10 µm can be measured. We hypothesize that while the nanofibrils of cellulose provide the support of the inner structure of hydrogel, the proteins backbone covers or entangles the nanofibers. From this standpoint, it is possible that the structural organization of the resulting scaffolds to be influenced by CNF without observing self-standing fibers. The interconnection of the pores is also an important parameter that defines the materials performance and the morphology of the nanofibrillar composite materials seems to be promising in terms of cell migration and proliferation as well as nutrients flow.

**Figure 4:**
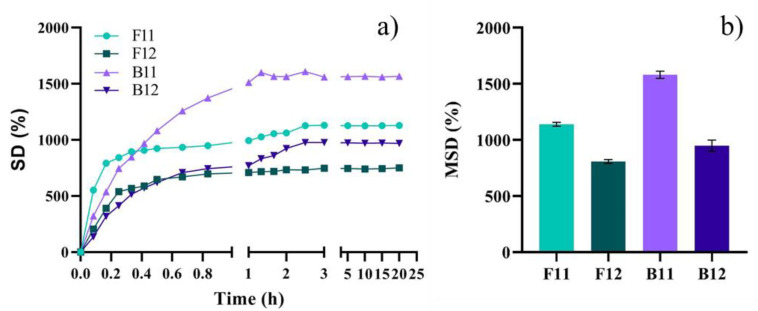
The swelling behavior of the freeze-dried printed scaffolds conducted in DMEM mimicking physiological conditions (pH 7.4 and 37 °C): rehydration kinetics (**a**) and maximum swelling degree (MSD%) (**b**).

The swelling behavior of scaffolds is conjectured by morphology and the assessment of the materials ability to rehydrate in aqueous media is essential when envisioning biomedical applications. Time-dependent swelling profiles along with the maximum swelling degree calculated for each sample are shown in Figure 4. To estimate the MSD, the samples were monitored for 24 h, during which it was observed that the materials remained stable. As a consequence of isotropic swelling, the scaffolds were capable of retaining their original shape after swelling. Throughout the free swelling process, the samples demonstrated that the material behaves in a geometry-independent manner, showing uniform dimensional change.

On the account of the CNF ability to absorb water up to 100 g/g water/dried mass, it was expected all samples to attain high swelling ratios [35]. Nevertheless, the crosslinking extent of GelMA network as well as the degree of hydrogen bonding and electrostatic repulsion between CNFs, GelMA and CNF-GelMA strongly influence the rehydration capacity of the nanocomposite biomaterials. The swelling kinetics display a fast water absorption within the very first minutes of immersion for all samples. Then, the scaffolds exhibited different swelling behavior with respect to GelMA type and CNF/GelMA ratio. The water absorption kinetics has generally shown a three-step rehydration curve: a fast rehydration in the first 10 min when up to 1/3 of the total amount of water is absorbed, a much slower rehydration for the next hour and then the SD% slightly increases again in the time frame of 3 h before reaching the equilibrium. The first rapid water uptake is attributed to the high scaffold’s porosity as the larger the pores are a higher amount of water is absorbed. The slow rehydration phase can be a result of the strong hydrogen bonding between the nanofibrils and thus the aqueous media requires longer time to infiltrate. Once the H-bonding is weakened, the hydrogel network can expand with respect to its crosslinking degree. However, the swelling profile behaved differently for F12 scaffolds, where a two-step curve was obtained. Similar to the rest of the samples, F12 samples undergo a rapid rehydration step within the first 20 min and then slowly reach the equilibrium after 1 h of immersion. Although it is expected that the addition of GelMA within CNF aqueous suspension to weaken the interactions between fiber bundles regardless the type and amount of protein, F-GelMA seems to express a higher affinity for CNF. In terms of MSD% it can be observed that the hydrogels with lower GelMA content exhibit higher swelling capacity: above 1000%. Since both F11 and B11 had presented morphologies with large pore size, a high-water uptake was expected. Increasing the amount of GelMA, the swelling rate decreases most probably due to a much compact morphology generated by a higher solid content and an increased number of crosslinking sites. With respect to the type of GelMA, it can be observed that F-GelMA scaffolds showed lower MSD values in comparison with B-GelMA samples. However, in line with a previous study wherein pristine hydrogels based on GelMA from fish gelatin and porcine gelatin are compared in terms of swelling behavior [12], it must be pointed out that our result is likely to be a a consequence of higher affinity of F-GelMA to CNF that is translated in ionic interaction in addition to H-bonding. Also, the binding of protein with CNF is considered to facilitate the crosslinking of GelMA [36]. The water absorption capacity of a hydrogel is an essential feature that may indirectly show how quickly it will degrade. It is critical to highlight that the swelling capacity and degradation rate of scaffolds are essential qualities of a successful hydrogel, since these characteristics influence healing. Several prior studies report, regardless of its source, that the two most significant drawbacks of the use of GelMA for tissue engineering are its poor mechanical characteristics and short degradation time, particularly for long-term implantation and for tissues that need substantial load-bearing capabilities [12,31,37]. Herein, our findings demonstrate that utilizing CNF in combination with F-GelMA proved to be beneficial in terms of swelling capacity. Yet, additional studies to understand more completely the key tenets of the degradation behaviour of GelMA-based formulation are required and should consider both the CNF impact and the 3D design and pore-size influence.

### 3.4. Evaluation of Cellular Response

#### 3.4.1. Acellular Scaffolds Biocompatibility

**Figure 5:**
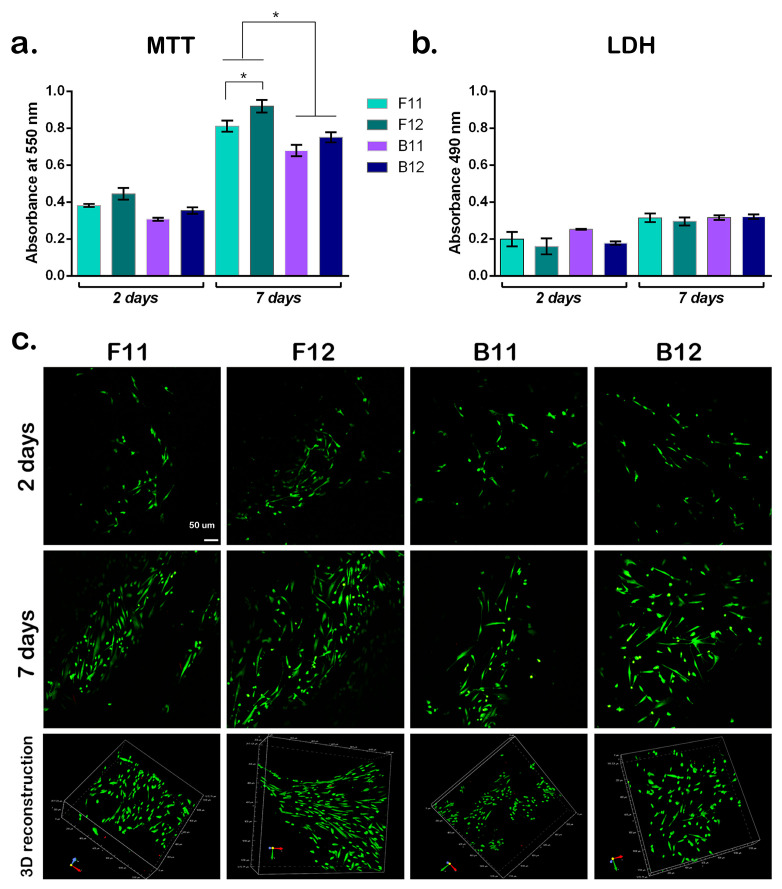
Biocompatibility evaluation of the acellular scaffolds. Cells were seeded on top of the scaffolds and biocompatibility was evaluated after 2 and 7 days of culture in standard conditions. (**a**) Cell viability and proliferation profiles obtained by MTT assay. Statistical significance: * *p* < 0.05. (**b**) GelMA-based scaffolds’ cytotoxicity profiles measured by LDH assay. (**c**) Qualitative evaluation of cell viability and proliferation was assessed employing Live/Dead assay and confocal microscopy. Live cells are stained in green fluorescence, while dead cells nuclei are stained in red fluorescence using LIVE/DEAD™ Viability/Cytotoxicity Kit, for mammalian cells (Thermo Fisher Scientific Inc., Waltham, MA, USA). Scale bar 50 µm.

Cell behavior and response is crucial for the development of effective scaffolds for tissue regeneration and given the anchorage-dependent character of cells, the scaffold design is consequential to the cell survival, adhesion, and proliferation. The cytocompatibility assessment of the bioengineered scaffolds printed as discs was performed using hASCs, considering their multi-lineage capacity that provides regeneration potential for various tissues. The biocompatibility evaluation of the acellular scaffolds after 2 and 7 days of culture is displayed in Figure 5. hASCs in direct contact with the printed scaffolds showed high viability rates in all samples after 2 days of culture, indicating that the combination of CNF and GelMA provide a proper environment to cells. The viability rate of hASCs was observed to increase with increasing the CNF/GelMA ratio from 1:10 to 1:20, given the higher prevalence of RGD sequences that promote adhesion and proliferation of cells. From 2 to 7 days of culture, cell viability increased on all composites, suggesting cell growth and proliferation in contact with 3D constructs. The assessment at 2- and 7-days post-seeding showed significant statistical differences with respect to the ratio between the two polymers and moreover, exhibited a statistically significant higher viability within F-GelMA samples with respect to B-GelMA specimens. As shown in Figure 5b, the number of cells on the F11 and B11 was significantly less than on F12 and B12 respectively. Also, significant differences in the number of cells were noticed among GelMAs from different sources since the proliferation rate of the cells is significantly higher on the F-GelMA compared to B-GelMA samples.

In addition to the biocompatible nature of the polymeric builders, the scaffolds were synthesized to have controlled architectures with highly porous structure that further has a significant influence over the cellular response. The F12 and B12 samples which exhibit smaller pores and larger specific surface areas (Figure 3f,h) registered higher cell viability and proliferation compared to F11 and B11. Cells developed spindle-like phenotype in contact with F12, B12, and F11, while in contact with B11 most of them maintained a rounded shape. These results showed that all samples displayed low rates of cytotoxicity and no significant cytotoxicity differences between compositions. Nonetheless, the best cell viability and cell distribution was found on F12 sample, indicating that this ink formulation is most suitable for growth and development of cells.

#### 3.4.2. Evaluation of Cell’s Viability toward 3D Biofabrication

**Figure 6:**
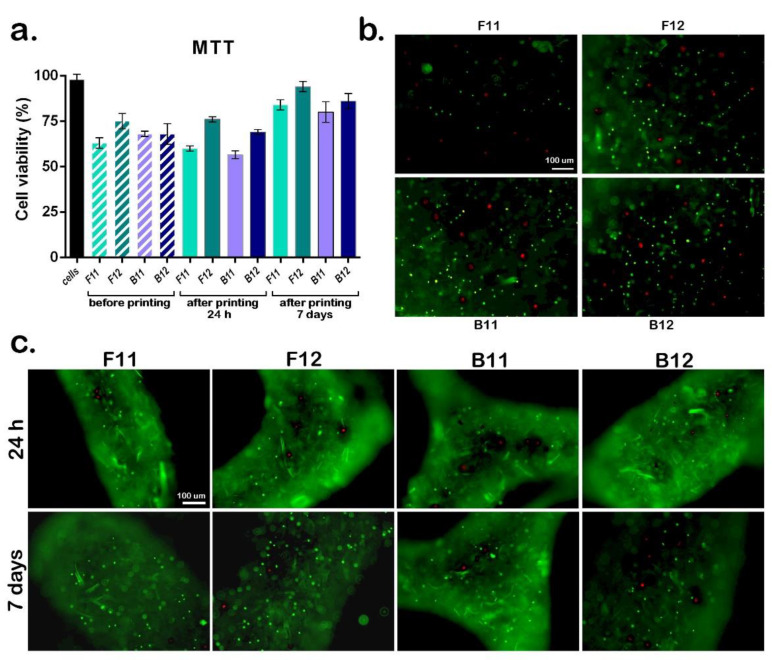
3D bioprinted scaffolds biocompatibility assessment. Quantitative evaluation of cell viability by MTT (**a**) showed comparatively viability of cells in culture, cell viability in bioink and cell viability in the bioprinted scaffolds after 24 h and 7 days of culture. LiveDead staining and confocal microscopy highlighted the ratio between live (green) and dead (red) cells before printing (**b**) and after printing (**c**) in 3D cultures exposed to standard conditions for 24 h and 7 days. Scale bar 100 μm.

Cell-laden 3D printed constructs are able to provide an enhanced biomimetic response since an improved arrangement between cells and the biomaterial support is ensured by the controlled the deposition of cell population in a 3D architecture. To evaluate the cell survival during the 3D biofabrication process, the newly developed biomaterial inks were embedded with hASCs and the cell viability was evaluated on bioink constructs. Figure 6 shows the assessment of scaffolds biofabrication towards cell viability in culture, in bioink and in the bioprinted scaffolds after 24 h and 7 days of culture. LiveDead staining and confocal microscopy were used to highlight the ratio between live and dead cells before printing and after printing in 3D cultures exposed to standard conditions for 24 h and 7 days. Investigation of cell viability revealed 98% cell viability in hASCs before embedding in comparison with cell viability after embedding in ink formulation and cross-linking the samples (~65–75% cell viability in non-printed specimens). Upon subjecting cell-embedded formulation to the printing process, no significant changes in the viability of cells within biofabricated scaffolds were observed (~62–75% cell viability in 3D printed specimens) compared with non-printed specimens at 24 h of culture. On the account that the number of live cells in the biomaterial ink before and after printing are comparable for each corresponding formulation after 24 h of incubation, and considering the cytocompatibility of the acellular scaffolds, the decrease in viability is most likely to be a preparation and mixing after-effect. Nonetheless, significantly higher viability was observed for samples with CNF/GelMA ratio of 1:20 compared to the ones with lower protein content. Also, in accordance with the biocompatibility assessment of acellular scaffolds, F-GelMA samples exhibit higher cell viability rates compared to B-GelMA samples before and after printing.

The evaluation of cell viability after 7 days of incubation revealed a statistically significant increase of cell proliferation rates for all samples, F-GelMA based samples presenting better results compared with B-GelMA. The cell viability constantly trends to increase with increasing the protein content and with respect to GelMA source. Among the formulated bioinks, F12 yielded increasingly good results exhibiting the highest viability rate upon cell-embedding, bioprinting and short-term 3D culture. Our results agree well with a previous study in which cells cultured in F-GelMA hydrogels demonstrated high cell viability and proliferation when compared with porcine GelMA [12]. While the same paper claims F-GelMA as a very promising component in bioinks for 3D printing applications, our research outcomes surpass prior findings. From these results it is clear that a proper environment for the loading, distribution, and proliferation of cells is provided by bioinks formulated using F-GelMA in combination with CNF, also as a consequence of a lower viscosity and thermal stability at room temperature of the biomaterial ink.

## 4. Conclusions

3D printing, the technology that sparked a prodigious development of manufacturing processes, evolved as the core technology of biofabrication and came along with new requirements in designing and combining biomaterials and cells into biological constructs. Thus, the development of suitable bioprinting materials became an important research topic where one of the central challenges is to obtain consistent, reliable, and cell-interactive materials for biofabrication. On these grounds, this research study succeeded to introduce for the first time highly reliable and stable bioinks based on GelMA in combination with CNF. By using gelatin from different sources (fish and bovine gelatin), biosynthetic inks were formulated and studied in comparison by systematically assessing the printability under cell appropriate conditions and following their overall functionality. The biosynthetic inks were formulated using fish- and bovine gelatin, both modified with methacrylamide side groups. These results showed that GelMA, which retains the cell-adherent motifs of collagen, in combination with CNF can render printable formulations. The physical interactions between the protein and polysaccharide proved to be beneficial to the material’s structural integrity, CNF backing gelatins printability. Throughout the fabrication process, the physical interactions between the components were conserved by photo-induced crosslinks and all formulations yielded 3D constructs with high shape fidelity and well-defined internal structure. Nonetheless, the bioprintability window is seen as a trade-off between ease of printing and biocompatibility. In the present study, we have shown that formulations based on F-GelMA are more readily to exceed the range of printability at low pressures and allow a uniform and continuous filament deposition regardless of increasing temperature, which translates in “easier to be printed”. As a result, it is fair to assert that the formulations based on fish gelatin exhibited a broader bioprintability window and thus F-GelMA proved to be a more reliable ink component in comparison to mammalian gelatin derivative as it can surmount the problems associated with significant viscosity changes upon temperature variation. Looking forward, these results provide a good starting point for further research toward the control of biomechanical properties of CNF/F-GelMA materials.

The results of the cytocompatibility assessment performed on acellular 3D scaffolds using hASCs, showed that the combination of CNF and GelMA provides a proper environment for cell growth and proliferation since high viability rates were determined for all samples during incubation. Comparing the results with respect to GelMAs source, evident differences in the number of cells were noticed. Higher cell viability and cell distribution were found on F-GelMA samples, especially when a higher protein concentration was employed, indicating that F12 ink formulation is most suitable for growth and development of cells. Moreover, the bioprinting tests demonstrated the ability of newly developed biomaterial inks to preserve the viability of embedded cells during the 3D fabrication process. Planned comparisons revealed that the cell viability constantly trends to increase when increasing the protein content, while with respect to the GelMA source, superior results were seen for F-GelMA samples. Among the designed bioinks, F12 exhibited from the beginning the highest viability rate upon embedding of cells, printing and short-term 3D culture, thus indicating that in addition to low viscosity and thermal stability at room temperature, F-GelMA in combination with CNF provides a proper support for the loading, distribution, and proliferation of cells.

## Figures and Tables

**Table 1:** Biomaterial ink formulations.

Ink Code	F-GelMA *w/v* %	B-GelMA *w/v* %
F11	11	-
F12	22	-
B11	-	11
B12	-	22

**Table 2:** Printing parameters for CNF/GelMA biomaterial ink formulations.

Ink Code	Pressure (kPa)	Feed Rate (mm/s)	Valve Opening Time, VOT (µs)	Printing Delay (ms)	Theoretical Diameter (mm)	Real Diameter (mm)
F11	40 ± 2	10	650	0	0.34	0.81 ± 0.03
F12	40 ± 2	10	800	0	0.34	0.72 ± 0.02
B11	50 ± 2	10	900	100	0.34	0.84 ± 0.03
B12	70 ± 2	10	1100	100	0.34	0.78 ± 0.05

## Data Availability

Data sharing is not applicable to this article.
