# Correction: Cernencu et al. 3D Bioprinting of Biosynthetic Nanocellulose-Filled GelMA Inks Highly Reliable for Soft Tissue-Oriented Constructs. *Materials* 2021, *14*, 4891

**DOI:** 10.3390/ma19050986

**Published:** 2026-03-04

**Authors:** Alexandra I. Cernencu, Adriana Lungu, Diana M. Dragusin, Izabela C. Stancu, Sorina Dinescu, Liliana R. Balahura, Paul Mereuta, Marieta Costache, Horia Iovu

**Affiliations:** 1 Advanced Polymer Materials Group, University Politehnica of Bucharest, 011061 Bucharest, Romania; alex.cernencu@gmail.com (A.I.C.); diana.m.dragusin@gmail.com (D.M.D.); izabela.stancu@upb.ro (I.C.S.); horia.iovu@upb.ro (H.I.); 2 Department of Biochemistry and Molecular Biology, University of Bucharest, 050095 Bucharest, Romania; sorina.dinescu@bio.unibuc.ro (S.D.); roxana.balahura@bio.unibuc.ro (L.R.B.); 3 Horia Hulubei—National Institute for Physics and Nuclear Engineering (IFIN-HH), 30 Reactorului Street, 077125 Magurele, Romania; paul.mereuta@gmail.com; 4 Academy of Romanian Scientists, 54 Splaiul Independentei, 050094 Bucharest, Romania

In the original publication [1], there was an overlap in Figure 5c as published. The corrected Figure 5c appears below. 

As Marieta Costache passed away in February 2024, a “†” symbol has been added to her affiliation, and her email address has been removed from this original publication [1].

The authors state that the scientific conclusions are unaffected. This correction was approved by the Academic Editor. The original publication has also been updated.

## Figures and Tables

**Figure 5:**
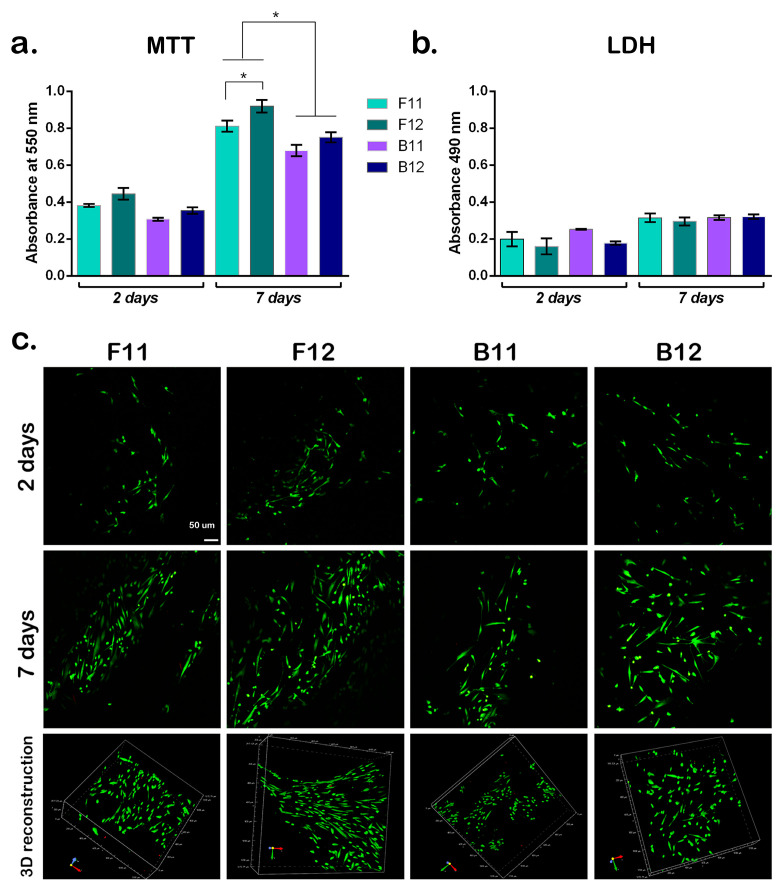
Biocompatibility evaluation of the acellular scaffolds. Cells were seeded on top of the scaffolds and biocompatibility was evaluated after 2 and 7 days of culture in standard conditions. (**a**) Cell viability and proliferation profiles obtained by MTT assay. Statistical significance: * *p* < 0.05. (**b**) GelMA-based scaffolds’ cytotoxicity profiles measured by LDH assay. (**c**) Qualitative evaluation of cell viability and proliferation was assessed employing Live/Dead assay and confocal microscopy. Live cells are stained in green fluorescence, while dead cells nuclei are stained in red fluorescence using LIVE/DEAD™ Viability/Cytotoxicity Kit, for mammalian cells (Thermo Fisher Scientific Inc., Waltham, MA, USA). Scale bar 50 µm.
